# Leaf apoplastic alkalization promotes transcription of the ABA-synthesizing enzyme Vp14 and stomatal closure in *Zea mays*

**DOI:** 10.1093/jxb/eraa589

**Published:** 2020-12-21

**Authors:** Christoph-Martin Geilfus, Xudong Zhang, Axel Mithöfer, Lisa Burgel, Gyöngyi Bárdos, Christian Zörb

**Affiliations:** 1 Division of Controlled Environment Horticulture, Albrecht Daniel Thaer-Institute of Agricultural and Horticultural Sciences, Humboldt-University of Berlin, Albrecht-Thaer-Weg, Berlin, Germany; 2 Institute of Crop Science, Quality of Plant Products, University Hohenheim, Schloss, Westhof-West, Stuttgart, Germany; 3 Max Planck Institute for Chemical Ecology, Research Group Plant Defense Physiology, Hans-Knöll-Straße, Jena, Germany; 4 Lancaster University, UK

**Keywords:** ABA, alkalinization, apoplast, chloride, guard cell, NCED, salinity, stomata, transpiration

## Abstract

The chloride component of NaCl salinity causes the leaf apoplast to transiently alkalinize. This transition in pH reduces stomatal aperture. However, whether this apoplastic pH (pH_apo_) transient initiates stomatal closure by interacting with other chloride stress-induced responses or whether the pH transient alone initiates stomatal closure is unknown. To clarify the problem, the transient alkalinization of the leaf apoplast was mimicked in intact maize (*Zea mays* L.) by infiltrating near-neutral pH buffers into the leaf apoplast. Effects of the pH_apo_ transient could thus be investigated independently from other chloride stress-derived effects. Microscopy-based ratiometric live pH_apo_ imaging was used to monitor pH_apo_*in planta*. LC-MS/MS and real-time quantitative reverse transcription–PCR leaf analyses showed that the artificially induced pH_apo_ transient led to an increase in the concentrations of the stomata-regulating plant hormone abscisic acid (ABA) and in transcripts of the key ABA-synthesizing gene *ZmVp14* in the leaf. Since stomatal aperture and stomatal conductance decreased according to pH_apo_, we conclude that the pH_apo_ transient alone initiates stomatal closure. Therefore, the functionality does not depend on interactions with other compounds induced by chloride stress. Overall, our data indicate that the pH of the leaf apoplast links chloride salinity with the control of stomatal aperture via effects exerted on the transcription of ABA.

## Teaser

In maize, the transient alkalinization of the leaf apoplast acts as a switch that increases transcription of the ABA-synthesizing key gene *viviparous 14* and reduces transpiration.

## Introduction

During soil water scarcity, the pH of the leaf apoplast (pH_apo_) is relevant for the regulation of the guard cell pore size. This is because leaf pH_apo_ is implicated in the root to shoot communication of progressive soil drying (Sobeih *et al.*, 2004; Wilkinson and Davies, 2008). A drought stress-induced increase in pH_apo_ links root zone drying with the regulation of the stomatal aperture by acting on the compartmental distribution of the guard cell-regulating phytohormone abscisic acid (ABA) between the leaf apoplast and symplast (Davies et al., 2002, 2005; Wilkinson and Davies, 2008).

There is, however, an ongoing debate over the site of synthesis of the ABA that accumulates in the leaf in response to soil water scarcity. Some studies conclude that the root is the major site of ABA biosynthesis during soil drying, with root-derived ABA acting as a long-distance chemical signal regulating stomatal conductance (Wilkinson, 1999; Wilkinson *et al.*, 2007; Boyle *et al.*, 2016). New knowledge gained from experiments on reciprocal grafts of ABA-deficient mutants and wild-type (WT) tomato (*Solanum lycopersicum*) plants has challenged this view. Reciprocal grafting experiments with ABA-synthesizing WT and ABA-deficient mutants revealed rootstock independence of stomatal regulation, irrespective of the ABA concentration delivered via the xylem from root to shoot (Holbrook *et al.*, 2002; Dodd *et al.*, 2009; Li *et al.*, 2018). These grafting experiments indicate that rootstock ABA synthesis seems to be less important in regulating leaf ABA concentration and stomatal conductance as the soil dries than originally assumed (Holbrook *et al.*, 2002; McAdam *et al.*, 2016*a*). Instead, a production of ABA in phloem companion cells of the vasculature (Kuromori *et al.*, 2014) might feed the apoplast of water-stressed leaves with ABA, as shown for water-stressed *Arabidopsis thaliana*. In addition, *A. thaliana* guard cell-autonomous ABA synthesis could allow the leaf to maintain hydration (Bauer *et al.*, 2013). In maize, the 9-*cis*-epoxycarotenoid dioxygenase (*NCED*) gene family ortholog *viviparous 14* (*Vp14*) encodes the rate-limiting enzyme in ABA biosynthesis (Seo and Koshiba, 2002; Xiong and Zhu, 2003). The *ZmVp14* gene was characterized from the ABA-deficient maize mutant *Vp14* (Tan *et al.*, 1997) and is known to be regulated by salinity and osmotic stress Geilfus *et al.*, 2018).

For the fraction of the ABA that is not synthesized directly within the guard cell, but has to overcome the leaf apoplastic passage towards the guard cells, the alkalinization of the leaf apoplast or xylem is believed to be important. Such drought stress-induced increases in leaf pH_apo_ and shoot xylem sap pH, respectively, have been shown for field bean (*Vicia faba* L.; Karuppanapandian *et al.*, 2017), maize (*Zea mays* L.; Bahrun *et al.*, 2002), sunflower (*Helianthus annuus* L.; Gollan *et al.*, 1992; Schurr *et al.*, 1992) Asiatic dayflower (*Commelina communis* L.; Wilkinson and Davies, 1997), grapevine (*Vitis vinifera* L.; Stoll *et al.*, 2000), tomato (*Solanum lycopersicum* L.; Wilkinson *et al.*, 1998), hops (*Humulus lupulus* L.; Korovetska *et al.*, 2014), and many others.

However, this drought-induced alkalinization is not universal in all plant species, which has cast doubt on the assumption that it is a ubiquitous long-distance signal that regulates stomatal responses to soil drying (Sharp and Davies, 2009). Of 22 woody perennial species from different plant families, only four (*Buddleja davidii*, *Dicksonia antarctica*, *Penstemon heterophyllus*, and *Rhododendron obtusum*) evolutionarily unrelated species alkalized their xylem sap as the soil dried, but these species showed the greatest control of water status during water scarcity (Sharp and Davies, 2009). Moreover, within eight herbaceous species from various taxonomic groups the ability to alkalinize xylem sap in response to soil drying was not universal, and unrelated to phylogeny (Gloser *et al.*, 2016). Overall, alkalinization was more likely in annual plant species than in woody perennial plants, but there are few insights as to why this is so.

Nevertheless, for those species that alkalinize the apoplast in response to soil drying, the following mode of action is proposed for regulating stomatal aperture. Under well-watered conditions, ABA is released into the leaf apoplast. The apoplastically located ABA is present in its undissociated form (ABAH) because it is a weak acid and because the apoplast is slightly acidic (Hartung, 2010). By following its chemical gradient, the uncharged ABAH diffuses from the leaf apoplast through the plasma membrane (PM) into mesophyll cells. As soon as ABAH enters the slightly alkaline cytosol of the mesophyll, the weak acid dissociates to ABA^−^ by releasing a proton (H^+^). The phytohormone is now charged (ABA^−^) and is trapped inside the mesophyll cells because charged compounds cannot diffuse across the PM (Hartung *et al.*, 1988). It is then stored or metabolized in the mesophyll ( Slovik and Hartung, 1992; Wilkinson and Davies, 1997).

However, during progressive soil drying, the pH of the leaf apoplast increases above the p*K*a of ABA (=4.87) (Karuppanapandian *et al.*, 2017). Thus, under conditions of water scarcity, the ABA that is increasingly delivered from the root or stem dissociates in the leaf apoplast into its charged anionic form, becoming trapped extracellularly (Wilkinson and Davies, 1997). Such a pH_apo_-based accumulation of ABA^−^ in the leaf apoplast was recently demonstrated under conditions of NaCl salinity when the chloride component of NaCl salinity provoked the leaf apoplast of the field bean to alkalinize over a period of 2–3 h, before it re-acidified once again (Geilfus *et al.*, 2015). After re-acidification of the leaf apoplast back to the initial steady-state pH, the guard cell-intrinsic ABA concentration was increased and the stomata were closed. The guard cell-intrinsic ABA concentration was proposed to rise because the sudden re-acidification of the leaf apoplastic compartment established such a high free proton concentration in the apoplast that the bulk of the apoplastically trapped ABA^−^ was associated with protons. In other words, a fraction of membrane-permeable ABAH was formed in the leaf apoplast in the vicinity of the guard cells; this ABAH followed its gradient and streamed into the guard cells. This is in good agreement with Goodger *et al.* (2005), who report for maize that the release rates of both ABA and chloride out of mesocotyl xylem sap that is flowing from roots into the shoot correlate with stomatal conductivity. We previously demonstrated that the chloride-induced transient leaf apoplastic alkalinization was instrumental in inducing stomatal closure during the beginning of NaCl salinity through pH_apo_-based effects on the compartmental distribution of ABA (Geilfus *et al.*, 2015). Experiments with different chloride- or sodium-accompanying counter ions, and with agents that mimicked osmotic stress, revealed that the reduction of the stomatal aperture was linked to the chloride component, namely to the chloride-induced transient leaf apoplastic alkalinization (Geilfus *et al.*, 2015). Nevertheless, stomatal aperture was not reduced when leaf concentrations of chloride increased during the experimental suppression of the formation of the pH_apo_ transient. This was established by clamping the pH_apo_ in the acid range by the infiltration of an acid pH buffer. Hence, the chloride-induced transient alkalinization of the leaf apoplast was inhibited, although chloride was taken up excessively into the leaf. These experiments showed that the pH_apo_ transient mechanistically links chloride stress with a reduction in stomatal aperture (Geilfus *et al.*, 2015).

Nevertheless, these experiments have not revealed whether the formation of the transient apoplastic alkalinization alone, (i) independently from other chloride stress-induced responses and (ii) in the absence of excessive uptake of chloride, is functional in initiating a reduction in stomatal aperture. Only an experimental set-up in which this transient leaf apoplastic alkalinization is mimicked but without stressing the plants with chloride is capable of clarifying this point. With this aim, the chloride-inducible alkalinization of the leaf apoplast was mimicked by infiltrating near-neutral pH buffers in this study. We hypothesized that the formation of this artificially induced pH_apo_ transient reduces stomatal aperture, despite the plants not being salinity stressed, and that ABA synthesis is under control of the pH_apo_ transient. To elaborate these hypotheses, the apoplastic pH dynamics, the transcript abundance of the ABA-synthesizing key enzyme ZmVp14, the abundance of ABA, the stomatal pore size, stomatal conductance (*g*_s_), and the transpiration rate (E) were quantified in the leaves of hydroponically gown maize.

## Materials and methods

### Experimental design

Maize (*Zea mays* L.) was cultivated hydroponically in nutrient solution (in 2019 and 2020). To mimic the formation of the transient alkalinization of the leaf apoplast in a way comparable (regarding duration and magnitude) with the chloride-induced alkalinization of the leaf apoplast (see Geilfus *et al.*, 2015), the pH buffer MES (5 mM, pH 6.5) was infiltrated into the leaf apoplast. Ratiometric real-time pH_apo_ imaging was applied to monitor the pH_apo_ transient *in planta* (see below). The pH buffer was mixed with the pH indicator dye Oregon Green (OG) 488-dextran (25 µM) before infiltration. A volume of 0.4 ml of the pH buffer/dye mixture was infiltrated using a needleless syringe. A second experimental group was set up to rule out unspecific effects that may have arisen from the buffering agent (i.e. effects that did not arise from the pH_apo_). For this purpose, 0.4 ml of a mixture of the pH buffer 2-hydroxy-MOPS (MOPSO) (7.5 mM, pH 6.5) and OG dye was infiltrated into the leaf. To guard against effects possibly arising from the infiltration procedure or the infiltration of water, control plants were infiltrated with 0.4 ml of a mixture of water and the OG dye. Osmotic controls were used to test for osmotic effects possibly arising from the pH buffer agents. For this, 0.4 ml of a mixture of the pH buffer MES (7.5 mM, pH 4.7) and OG dye was infiltrated into the leaf. In this fourth experimental group, the pH was set to 4.7 because this proton concentration prevails in the leaf apoplast.

In total, four experimental groups were tested. (i) Control: infiltration of 0.4 ml of an aqueous 25 µM OG solution. (ii) Osmotic control: infiltration of 0.4 ml of a mixture of 7.5 mM MES (pH 4.7) and 25 µM OG. (iii) MES buffer: infiltration of 0.4 ml of a mixture of 5 mM MES (pH 6.5) and 25 µM OG. (iv) MOPSO buffer: infiltration of 0.4 ml of a mixture of 7.5 mM MOPSO (pH 6.5) and 25 µM OG.

To investigate effects of the formation of the artificially induced pH_apo_ transient on the transcription of the ABA-synthesizing maize gene *9-cis-epoxycarotenoid dioxygenase 1* (*ZmVp14*; Tan *et al.*, 2003) and on the abundance of ABA, leaves were sampled at eight key time points to reflect the situation before, during, and after the alkalinization. Microscopy-based live pH_apo_ imaging allowed the identification of these eight time points. A separate batch of plants was necessary for sampling leaf material at each time point. Sampled leaf material was immediately frozen in liquid nitrogen. Since pH_apo_ imaging was performed *in planta* by using a size-calibrated microscope, the size of the stomatal aperture could be quantified concurrently. Transpiration was measured in parallel on separate batches of plants that were treated identically. To confirm that one-time infiltration of 0.4 ml MES or the MOPSO buffers does not disturb the leaf in the long term, we checked rates of photosynthesis and transpiration at 24, 48, and 72 h after infiltration (Supplementary Table S1). For all measurements, the number of biological replicates varied between four and six, as indicated in the figure legends or table captions. Biological replicates represented individual plants taken from different cultivations.

### Plant cultivation

*Zea mays* (cv. Susann, Nordsaat Saatzucht GmbH, Langenstein, Germany) was cultivated in a hydroponic system using a controlled-environment chamber. Seeds were soaked for 1 d before being placed in moistened quartz sand for germination. Four days later, seedlings were transferred into 5 litre plastic pots containing one-quarter strength nutrient solution. After 2 d, the nutrient concentration was increased to half-strength and, after 4 d, to full-strength. The solution was changed every 3.5 d to avoid nutrient depletion. The nutrient solution had the following composition: 1.0 mM K_2_SO_4_, 2.5 mM Ca(NO_3_)_2_, 0.6 mM MgSO_4_, 0.2 mM KH_2_PO_4_, 1.0 mM CaCl_2_, 0.01 mM NaCl, 1.0 µM H_3_BO_4_, 2.0 µM MnSO_4_, 0.3 µM CuSO_4_, 0.5 µM ZnSO_4_, 200 µM Fe-EDTA, 0.005 µM (NH_4_)6Mo_7_O_24_. Plants were cultivated under a 14 h (20 °C):10 h (18 °C) light:dark cycle (photoperiod 08.00–22.00 h) with an atmospheric water vapour pressure deficit (VPD) of 0.58 kPa (75% relative humidity) during the photoperiod. Light intensity was 500 μmol s^−1^ m^−2^ above the leaf canopy. Plants grew for 10 d in full-strength nutrient solution before the pH_apo_ transient was induced by infiltration of the pH buffer.

### Quantification of leaf apoplastic pH and determination of stomatal aperture

Leaf pH_apo_ and stomatal aperture were quantified using a calibrated Leica microscope (Leica DMi8; Leica Microsystems, Wetzlar, Germany). The entire plant was located in a cage incubator system, which allowed the precise control of the VDP (0.58 kPa; 75% relative humidity at 20 °C), and white light illumination via an LED over the entire experiment [light intensity was 400 µmol m^–^ s^–1^ of photosynthetic photon flux density (PPFD)]. LED illumination was automatically switched off during fluorescence image acquisition for pH_apo_ quantification. For the *in vivo* quantification of leaf pH_apo_, a 25 μM solution of the fluorescent pH indicator dye OG 488-dextran (Thermo Fisher Scientific, Darmstadt, Germany) was infiltrated into the leaf apoplast of intact plants by using a needleless syringe (Geilfus and Mühling, 2011), together with a pH buffer. where applicable. OG 488-dextran is a sensor for pH_apo_ because it does not enter the symplast, as shown by confocal imaging. Fluorescence images for calculation of pH_apo_ were collected as a time series with the Leica DMi8 microscope connected to a cooled sCMOS camera (Leica DFC9000 GT; Leica Microsystems). An HXP lamp (HXP Short Arc Lamp; Osram) was used for illumination at the excitation wavelengths of 440/10 nm and 490/10 nm. The exposure time was 25 ms for both channels. Emission was collected at 535/25 nm by using a band-pass filter in combination with a dichromatic mirror. The fluorescence ratio *F*_490_/*F*_440_ was obtained as a measure of pH_apo_ on a pixel-by-pixel basis. For conversion of the fluorescence ratio data into pH_apo_ values, an *in vivo* calibration was conducted as described elsewhere (Geilfus and Mühling, 2013). Stomatal aperture was quantified off-line on captured images by using the calibrated microscope.

### RNA extraction, cDNA synthesis, and real-time quantitative RT–PCR

Forward (f 5'–3': TTCTCGGAGGAGGAACAGAGGA) and reverse (r 5'–3′: CCAACTGTAACTCTGGTGTGCG) primers for amplifying *ZmVp14* mRNA were taken from Geilfus *et al.* (2018). For cDNA synthesis, RNA was isolated from 100 mg of ground lyophilized leaf material by using phenol–chloroform extraction according to the method of Cox and Goldberg (1988). The quality of RNA was checked by OD_260_ and OD_280_. A 1 µg aliquot of total RNA was digested with PerfeCTa DNAseI (Quanta Biosciences, Beverly, MA, USA) to eliminate residual genomic DNA, and reverse transcribed to single-stranded cDNA with the SuperScript VILO cDNA Synthesis Kit (Invitrogen by Life Technologies, Karlsruhe, Germany) according to the manufacturer’s instructions. The SYBR-Green-based real-time quantitative reverse transcription–PCR (real-time qRT–PCR) technique was performed on a BioRad CFX96 real-time PCR system by using iTaq Universal SYBR Green Supermix (BioRad Laboratories, Inc.). For each reaction, 0.8 µl of diluted single-stranded cDNA was used in a total volume of 10 µl (0.5 µM forward and reverse primer, 5 µl of iTaq Universal SYBR Green Supermix, topped up with sterilized autoclaved H_2_O_bidest_). Cycle information with regard to temperature, time, and cycle number is given in Geilfus *et al.* (2018). The specificity of the annealing was checked by dissociation kinetics performed at the end of the experiment (65–95 °C). The comparative ∆∆Ct (threshold cycles) method for relative quantification was used to analyse the data according to Pfaffl (2001). C_t_ values were normalized by comparison with the two endogenous reference genes actin 1 and ubiquitin-conjugating enzyme. Data are shown as the relative fold changes in transcript expression. Negative controls were carried out without templates. The specificity of the primer–template interactions was demonstrated by sequencing the real-time qRT–PCR products (GATC Biotech, Konstanz, Germany) (Supplementary Table S2). Moreover, agarose gels were run after real-time qRT–PCR to ensure that only a single PCR product was generated and to confirm the predicted PCR product size on the gel (Supplementary Fig. S1).

### ABA quantification

Extraction of ABA from the maize leaf was performed by adding 1 ml of extraction solution {5 ng of [^2^H_6_] (+)-*cis*,*trans* ABA internal standard (Olomouc, Czech Republic) in 7:3 methanol:water} to 10 mg of dried ground material. After being shaken for 30 min, samples were centrifuged at 16 000 *g* at 4 °C for 5 min. The supernatant was collected and dried under reduced pressure, re-dissolved in methanol (10 µl per 1 mg DW), mixed, and centrifuged at 16 000 *g* and 4 °C for 10 min. The supernatant was used for the LC-MS/MS-based analysis of ABA (Almeida Trapp *et al.*, 2014).

### Gas exchange measurements

A portable gas exchange system (LI-COR 6400 XT; LI-COR) was used to measure *g*_s_ (mmol H_2_O m^–2^ s^–^) and to obtain rates for E (mmol H_2_O m^–2^ s^–1^) and photosynthesis (μmol CO_2_ m^–2^ s^–1^). PPFD was 500 μmol m^−2^ s^−1^ as provided by the red, green, and blue LEDs of the integrated fluorescence chamber head (6400-02B LED light source; LI-COR). The leaf area included in the chamber was recorded for each leaf. VPD was 0.58 kPa (75% relative humidity; 20 °C). CO_2_ at a flow rate of 300 μmol mol^−1^ CO_2_ was controlled by a CO_2_ injection system.

## Results

### Infiltrating pH buffers set to pH 6.5 mimics a transient alkalinization of the leaf apoplast

**Fig. 1.**
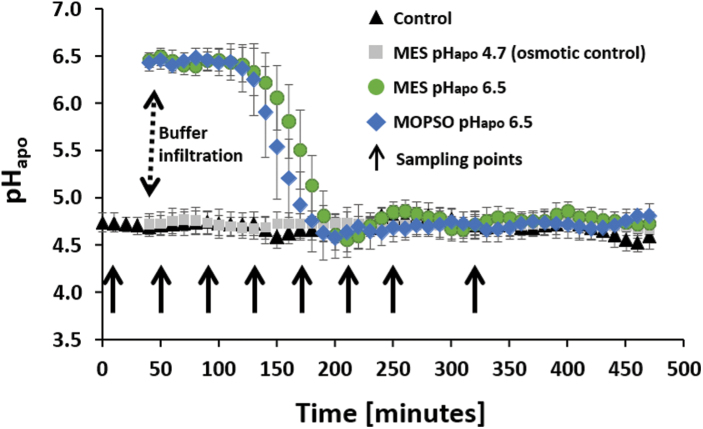
Infiltration of near-neutral pH buffers into the leaf apoplast results in a transient apoplastic alkalinization. Apoplastic pH as plotted over time. Green circles, infiltration of 0.4 ml of a mixture of 5 mM MES at pH 6.5 and 25 µM OG; blue diamonds, infiltration of 0.4 ml of a mixture of 7.5 mM MOPSO at pH 6.5 and 25 µM OG; black triangles, infiltration of 0.4 ml of an aqueous 25 µM OG solution (controls); grey squares, infiltration of 0.4 ml of a mixture of 7.5 mM MES at pH 4.7 and 25 µM OG (osmotic controls). Dashed black arrow, time point of infiltration; solid arrows, time points of the sampling of leaf material for subsequent *vp14* mRNA and ABA quantification. Representative pH kinetics of six equivalent recordings of leaves determined from independent experiments (*n*=6 biological replicates). Quantitation was technically averaged with *n*=4 regions of interest per ratio image and time point (shown as ±SD).

Infiltrating 0.4 ml of a 5 mM MES solution that was set to a pH of 6.5 into the leaf apoplast immediately increased the leaf pH_apo_ from 4.7 to 6.5 (Fig. 1, green circles). After remaining at pH 6.5 for a period of 80 min, the pH_apo_ started to re-acidify back to the initial steady-state pH of 4.7 over a period of 60 min. A similar transient alkalinization of the leaf apoplast was seen when 0.4 ml of a 7.5 mM MOPSO solution set to a pH of 6.5 was infiltrated into the leaf apoplast (blue diamonds). Control plants that were infiltrated with 0.4 ml of water showed a stable pH_apo_ of 4.7 over the entire experiment (black triangles). The pH_apo_ was also stable at 4.7 when 0.4 ml of a 7.5 mM MES solution set to a pH of 4.7 was infiltrated into the leaf (grey squares). The latter experimental group served as a control to exclude osmotic effects possibly arising from the infiltration of buffering agents (Fig. 1).

### Transient alkalinization of leaf apoplast increases abundance of *Vp14* mRNA

**Fig. 2.**
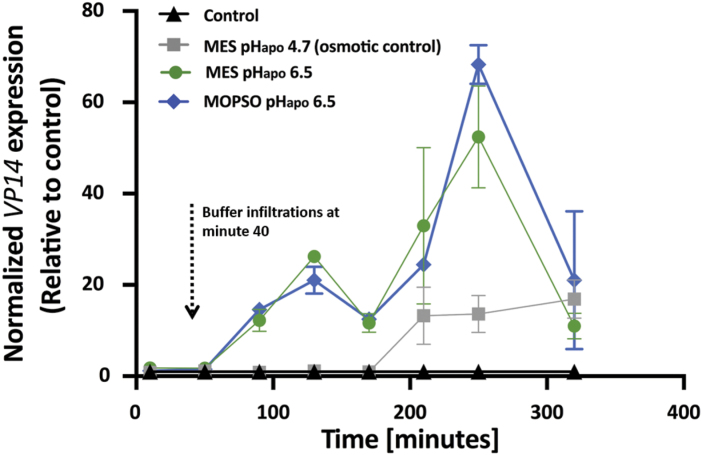
Transient alkalinization of the leaf apoplast increases mRNA abundance of *viviparous 14* (*Vp14*). Increases in transcript abundance shown as fold changes, relative to the control group (being set to 1). Green circles, infiltration of 0.4 ml of a mixture of 5 mM MES at pH 6.5 and 25 µM OG; blue diamonds, infiltration of 0.4 ml of a mixture of 7.5 mM MOPSO at pH 6.5 and 25 µM OG; black triangles, infiltration of 0.4 ml of an aqueous 25 µM OG solution (controls); grey squares, infiltration of 0.4 ml of a mixture of 7.5 mM MES at pH 4.7 and 25 µM OG (osmotic controls). Mean ±SE of four independent (*n*=4) biological replications (technically replicated in triplicate).

Infiltration of either MES or MOPSO buffer solutions each set to a pH of 6.5 into the leaf apoplast resulted in a steady increase of the abundance of *Vp14* mRNA, relative to control leaves (Fig. 2). At 210 min after buffer infiltrations (this time point is shown at minute 250 on the *x*-axis), the increases were the highest (52-fold in response to infiltration with MES at pH 6.5 and 68-fold in response to infiltration with MOPSO at pH 6.5). Subsequently, the alkalinization-induced increase in *Vp14* mRNA abundance decreased. Infiltration of the MES buffer set to a pH of 4.7 (osmotic control) resulted in a slight increase of *Vp14* mRNA abundance. In contrast to the alkalinization-induced increase, the osmotically caused increase was detected 120 min later, reaching a much lower maximum that occurred at 280 min after buffer infiltrations (this time point is shown at minute 320 on the *x*-axis) (Fig. 2).

### Leaf pH_apo_ transient causes leaf ABA concentrations to increase

**Fig. 3.**
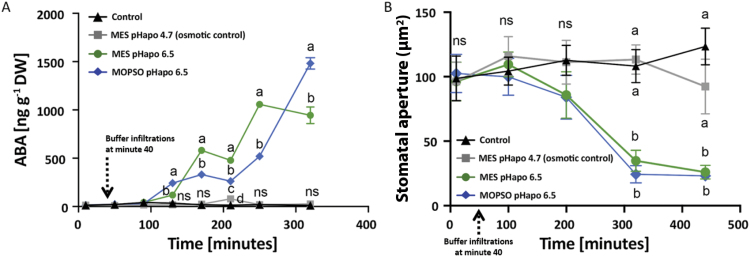
Artificially induced pH_apo_ transient increases leaf ABA concentration and reduces stomatal aperture. (A) Leaf ABA concentration; (B) stomatal aperture. Green circles, infiltration of 0.4 ml of a mixture of 5 mM MES at pH 6.5 and 25 µM OG; blue diamonds, infiltration of 0.4 ml of a mixture of 5 mM MOPSO at pH 6.5 and 25 µM OG; black triangles, infiltration of 0.4 ml of an aqueous 25 µM OG solution (controls); grey squares, infiltration of 0.4 ml of a mixture of 7.5 mM MES at pH 4.7 and 25 µM OG (osmotic controls). For ABA data: mean ±SE of four independent (*n*=4) biological replications (technically replicated in triplicate). Statistical significance (*P*≤0.05) between groups per time point as indicated by Tukey HSD test is shown by letters. For stomatal aperture data: mean ±SD of six independent (*n*=6) biological replications. Statistical significance (*P*≤0.05) between groups per time point as indicated by Tukey HSD test is shown by letters.

Infiltration of either MES or MOPSO buffer solutions each set to a pH of 6.5 into the leaf apoplast resulted in a steady increase of the leaf ABA concentrations, whereas the ABA concentration in the control leaves that were only infiltrated with water remained stable at 20±2 (mean ±SE) ng ABA g^–1^ DW over the entire experiment (Fig. 3A). At 280 min after buffer infiltrations (this time point is shown at minute 320 on the *x*-axis), the increases were at their highest (943±86 ng ABA g^–1^ DW in response to infiltration with MES at pH 6.5 and 1481±59 ng ABA g^–1^ DW in response to infiltration with MOPSO at pH 6.5). Infiltration of MES buffer set to a pH of 4.7 (osmotic control) did not cause a significant increase in ABA concentration (Fig. 3).

### Transient leaf apoplastic alkalinization reduces stomatal aperture, stomatal conductance, transpiration, and photosynthesis

At 60 min after infiltration of the pH buffers (this time point is shown at minute 100 on the *x*-axis of Fig. 1) or immediately after the re-acidification (this time point is shown at minute 200 on the *x*-axis of Fig. 1), neither the stomatal aperture (Fig. 3B) nor the stomatal conductance or the transpiration rate (Supplementary Table S3) was influenced by the infiltration of the MES and MOPSO buffer solutions set to a pH of 6.5 (when compared with the control that was infiltrated with water). At 2 h after the apoplast had re-acidified to a pH of 4.7 in response to the infiltration of the MES buffer at pH 6.5 (this time point is shown at minute 320 on the *x*-axis of Fig. 1), however, both the stomatal conductance (128±25 mmol H_2_O m^–2^ s^–1^) and the transpiration rate (2.3±0.4 mmol H_2_O m^–2^ s^–1^) were significantly lower compared with plants from the control group. In controls, *g*_s_ mmol H_2_O m^–2^ s^–1^ was 283±45 and E was 5.8±1.1 mmol H_2_O m^–2^ s^–1^, (Supplementary Table S3). At the same time point, the stomatal aperture significantly decreased from 108.2±12.8 µm^2^ to 34.8±8.1 µm^2^ in response to the MES-induced apoplastic alkalinization (Fig. 3B). Thus, it was significantly lower compared with the control. At 4 h after re-acidification (this time point is shown at minute 440 on the *x*-axis of Fig. 1), the transpiration rate (1.8±0.3 mmol H_2_O m^–2^ s^–1^), the stomatal conductance (97±26 mmol H_2_O m^–2^ s^–1^), and the stomatal aperture (26.0±5.3 µm^2^) were still significantly lower in plants from the group ‘MES pH_apo_ 6.5’ when compared with plants from the control (in the control, E was 6.1±0.9 mmol H_2_O m^–2^ s^–1^, *g*_s_ was 294±48 mmol H_2_O m^–2^ s^–1^, and stomatal aperture was 123.4±14.2 µm^2^). Similar responses were observed when the leaf apoplast was transiently alkalinized by using the MOPSO buffer set to pH 6.5, when compared with the control. The transpiration rate significantly decreased to 1.7±0.1 mmol H_2_O m^–2^ s^–1^ or 1.9±0.3 mmol H_2_O m^–2^ s^–1^ at 2 h or 4 h after re-acidification. Stomatal conductance significantly decreased to 85.3±14 mmol H_2_O m^–2^ s^–1^ or 102.26±26 mmol H_2_O m^–2^ s^–1^ at 2 h or 4 h after re-acidification (Supplementary Table S3). Stomatal aperture significantly decreased to 24.3±6.7 µm^2^ or 23.1±4.4 µm^2^ at 2 h or 4 h after re-acidification (Fig. 3B). In good agreement with the stomatal closure that was elicited by the apoplastic alkalinization, the rate of photosynthesis also decreased in response to the alkalinization at the same time points (Supplementary Table S3). Use of osmotic controls revealed that all these effects were not related to the osmotic properties of the buffer solutions: infiltration of a MES buffer set to a pH of 4.5 did not significantly affect stomatal aperture (Fig. 3B) or stomatal conductance and rate of transpiration (Supplementary Table S3).

### The pH_apo_-induced increase in ABA correlates positively with the decrease in *g*_s_

**Fig. 4.**
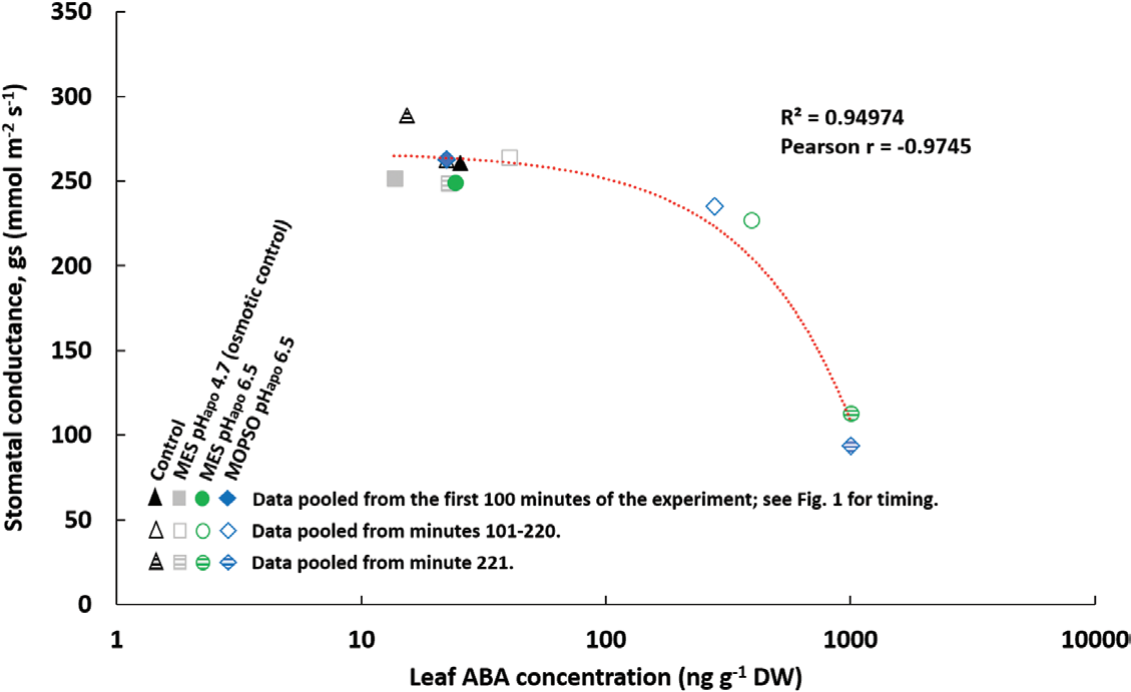
Correlation analysis of stomatal conductance and leaf ABA concentration. Circles, infiltration of 0.4 ml of a mixture of 5 mM MES at pH 6.5 and 25 µM OG; diamonds, infiltration of 0.4 ml of a mixture of 5 mM MOPSO at pH 6.5 and 25 µM OG; triangles, infiltration of 0.4 ml of an aqueous 25 µM OG solution (controls); squares, infiltration of 0.4 ml of a mixture of 7.5 mM MES at pH 4.7 and 25 µM OG (osmotic controls). Filled symbols, data were collected and pooled from the first 100 min of the experiment (please see *x*-axis in Fig. 1 to relate this phase to the leaf apoplastic pH); open symbols, data were collected and pooled from minutes 101 to 220; dashed symbols, data were collected and pooled from minute 221. Results of correlation analysis are indicated by the Pearson correlation coefficient *r*.

The Pearson correlation coefficient shows a strong statistical linear relationship between the increasing leaf ABA concentration and the decreasing stomatal conductance only when the apoplast was alkalinized (Pearson *r*= –82 for ‘MES pH_apo_ 6.5’; Pearson *r*= –95 for ‘MOPSO pH_apo_ 6.5’; data not shown). There was a much weaker (Pearson *r*= –59) or no (Pearson *r*=0.06) statistical relationship between both parameters in plants of the osmotic control or the water control, respectively (data not shown). The pH_apo_ effect on both stomatal conductance and ABA concentration becomes evident when fitting a single relationship between data points from all four experimental groups: Pearson *r* is –97 (Fig. 4) whereby plants from experimental groups with a re-acidified leaf apoplast (‘MES pH_apo_ 6.5’, ‘MOPSO pH_apo_ 6.5’) clearly clustered away from groups where pH_apo_ did not change over the experiment (‘control’ or ‘osmotic control”).

## Discussion

Soil salinization is a big constraint for plants because, among other mechanisms, it reduces the availability of water in the soil. Thus, salt and water stress have much in common (Munns, 2002). To maintain turgor and tissue hydration, the plant adjusts its transpiration. With regard to this, the pH of the apoplast is considered to act as a long-distance signal that transmits information about the decreasing soil water availability from root to shoot (Wilkinson, 1999; Wilkinson and Davies, 2008; Geilfus *et al.*, 2015). Upon arrival in the shoot, it regulates stomatal aperture by acting on the compartmental distribution of ABA between the shoot apoplast and symplast (Wilkinson, 1999; Davies *et al.*, 2002). In addition to this Henderson–Hasselbalch-regulated partitioning of ABA (Sharp and Davies, 2009), the present study demonstrates for the first time that the stomatal closure, elicited by the apoplastic alkalinization, is also due to a pH_apo_-mediated increase in the transcription of the NCED gene *Vp14* in the leaf. *ZmVp14* encodes the rate-limiting enzyme in ABA biosynthesis (Seo and Koshiba, 2002).

First of all, the study aimed at clarifying whether this leaf pH_apo_ transient is only functional with regard to the initiation of stomatal closure when interacting in concert with chloride ions or other chloride stress-related responses or variables that are induced by chloride salinity. These could include changes in [Ca^2+^], [H_2_O_2_], [ATP], or membrane potential. Following the concept of network signalling (Jordan, *et al.*, 2000; Plieth, 2016), all these variables (signals) would be integrated at downstream junctions to modulate the output (i.e. stomatal closure). Alternatively, the pH_apo_ transient alone might initiate a cascade of events that regulates aperture following the concept of single-file signalling (see Plieth, 2016). The latter proposition suggests that the pH_apo_ transient acts independently from other chloride stress-associated responses that are induced by chloride salinity.

To clarify this, we mimicked the transient alkalinization of the leaf apoplast, as occurs under conditions of chloridestress (of course, without adding chloride stress), by infiltrating near-neutral pH buffers (pH 6.5) into the leaf apoplast. By these means, we investigated the effect of the pH_apo_ transient independently from any chloride stress-derived responses; that is, unrelated to the excessive accumulation of chloride (or its accompanying cation). If the hypothesis that the pH_apo_ transient alone is able to initiate stomatal closure without interacting with other chloride stress-related responses is true, then we would expect that guard cell aperture and stomatal conductance would decrease in response to mimicking the chloride-induced pH_apo_ transient.

### pH_apo_ transient increases transcript abundance of the *Vp14* gene in maize leaves

To stimulate transient leaf apoplastic alkalinization in a similar way to that observed for chloride salinity, a MES-based pH buffer (pH 6.5) was infiltrated into the maize leaf apoplast (Fig. 1). To link this mimicked pH_apo_ dynamic with the synthesis of ABA, the transcript abundance of the ABA-synthesizing gene *ZmVp14* (Tan *et al.*, 2003) was analysed. Relative to the control, the *ZmVp14* transcript abundance increased by up to 52-fold (Fig. 2). To demonstrate that this effect was due to changes in the pH_apo_ and not from the buffering agent MES, a different buffer (MOPSO set to pH 6.5) was infiltrated in a repeat experiment. Relative to the control, *ZmVp14* transcript abundance increased by up to 68-fold (Fig. 2). Infiltration of an osmotic control (i.e. 7.5 mM MES solution that was set to a pH of 4.7) showed that it is the apoplastic pH transient that is responsible for the fast and steep increase in the transcription of the *Vp14* gene in maize, being unrelated to the osmotic properties of the buffer agents (Fig. 2). Any mechanical effects arising from the infiltration procedure or from flooding the apoplast with water can be excluded because controls were infiltrated with water and the above-mentioned fold changes were expressed relative to these controls. It has long been assumed that xylem-transported ABA, which was synthesized in the roots, is of major relevance for the shoot ABA pool when the plants experiences salt stress at the roots. The presented data on *Vp14* gene transcription demonstrated that the leaf is the key site of ABA synthesis, challenging the paradigm that the vast amount of the foliar-located ABA is produced in the roots. Reciprocal grafting experiments with ABA biosynthetic mutant and WT plants suggest that there is a signal that carries the information of a declining root water potential through the xylem to the leaves (Holbrook *et al.*, 2002; Christmann *et al.*, 2007). Upon arrival, leaf hydration, leaf ABA status, and stomatal aperture are adjusted (Manzi *et al.*, 2015; McAdam et al., 2016*a*, b, c). The leaf apoplastic alkalinization, that travels systemically and acropetally through the shoot (Geilfus *et al.*, 2015), appears to be a candidate for transmitting information about the onset of water scarcity because it induces leaf ABA synthesis (Figs 3, 4) and elicits stomatal closure (Supplementary Table S3).

### Transient apoplastic alkalinization modulates ABA and increases it in maize leaves

Next, we investigated whether the artificially induced leaf apoplastic alkalinization was associated with a rise in leaf ABA concentration. A shift in leaf apoplastic pH to 6.5 by means of MES and MOPSO infiltration caused the ABA concentrations steadily to increase steadily over the entire experiment relative to the water control and the osmotic control (Fig. 3). A temporal comparison (compare Fig. 2 and Fig. 3A) reveals that the increase of the *Vp14* mRNA abundance preceded the increase of the ABA concentration. This indicates that the increase in leaf ABA was caused by increased biosynthesis. However, the means by which the pH_apo_ transient is associated with *Vp14* expression awaits clarification. Is a pH sensor involved? Such a putative sensor would need a pH-responsive (apoplastic-located) domain as part of an integral protein that spans the entirety of the PM. It would sense changes in the pH_apo_ or the transmembrane pH gradient, passing on the information to start signal transduction via intermediate cellular transmitters that finally involve *Vp14* promoter elements. Because the existence of such a sensor is unclear, the elucidation of this problem is extremely difficult. Thus, other explanations have to be taken into consideration. For instance, large changes in the pH_apo_ are known to be able to influence the cytosolic pH (Monshausen *et al.*, 2007). Since cytosolic pH dynamics are accompanied by cellular Ca^2+^ transients (Monshausen et al., 2008, 2009; Michard *et al.*, 2016; Dindas *et al.*, 2018) and have been implicated in the specificity of Ca^2+^ signalling (Behera *et al.*, 2018), large transients in the pH_apo_ might affect ABA transcription via cellular signal transduction. However, such a sequence of events remains speculative unless a clear link between pH_apo_ transients, Ca^2+^ transients, and the induction of *Vp14* gene expression is established. Currently, the transcription of the *NCED* gene is known to be regulated by light (study on tomato by Thompson *et al.*, 2000), salinity and osmotic stress (study on maize by Geilfus *et al.*, 2018), water deficit (study on maize by Tan *et al.*, 1997), and drought (study on *Citrus* by Agustí *et al.*, 2007).

### Apoplastic alkalinization reduces stomatal aperture and transpiration rate

Wilkinson and Davies (2008) treated leaves of an ABA-deficient tomato mutant with alkaline-buffered foliar sprays and demonstrated that an increase in the leaf pH_apo_ reduced stomatal conductance only when ABA was simultaneously sprayed onto the leaves of the ABA-deficient tomato. They and others postulated that the apoplastic alkalinization required ABA to act on stomatal aperture (Wilkinson and Davies, 1997, 2008; Sobeih *et al.*, 2004; Else *et al.*, 2006; Jia and Davies, 2007) and suggested a pH_apo_-based mechanism whereby the rise in pH_apo_ increased the amount of ABA that penetrated into the guard cells by changing the compartmental distribution of ABA between the apoplast, the symplast, and various symplastic components.

The presented study adds knowledge about this system as it demonstrates that it is indeed the leaf apoplastic alkalinization that induces *Vp14* gene expression (Fig. 2), presenting a further mechanism that causes the foliar ABA level to rise (Fig. 3A) and stomatal conductance to decline (Fig. 4). This novel finding provides a compelling explanation for the observed stomatal closure and the decline in photosynthetic rate (Supplementary Table S3) elicited by the apoplastic alkalinization.

Apoplastic alkalinization can also be argued to induce stomatal closure directly via pH_apo_-based effects on guard cell K^+^ fluxes. A rising pH in the stomatal cavity decreases the activity of guard cell-localized inwardly rectified K^+^ channels and increases the activity of guard cell-localized outwardly rectified K^+^ channels (Hedrich *et al.*, 1995; Ache *et al.*, 2000). However, two arguments oppose the assumption that stomata close because of the direct pH_apo_-based stimulation of outwardly rectified guard cell K^+^ channels, highlighting the role of a pH_apo_-based effect of ABA biosynthesis. First, both the stomatal aperture and the transpiration rate remained reduced at 4 h after re-acidification (Supplementary Table S3). During these 4 h, the plants were continuously illuminated with white light. As light increases the activity of inwardly rectified K^+^ channels (Roelfsema and Hedrich, 2005; Marten *et al.*, 2007), enough time would have been available for a light-induced K^+^ influx to mediate the re-opening of the stomata. However, the stomatal aperture remained reduced. Second, the stomatal aperture and transpiration remained reduced, although the apoplast had become acidified once again, over the 4 h, to a pH_apo_ range that favoured K^+^ influx into guard cells via inwardly rectified K^+^ channels; such changes could ultimately result in guard cell re-opening, if not blocked by ABA.

### Conclusions

We have shown that an artificially induced transient of the pH of the leaf apoplast initiates stomatal closure and reduces the rate of leaf transpiration (Supplementary Table S3). A pH_apo_-based *de novo* synthesis of the guard cell-regulating hormone ABA in leaves (Fig. 2) and a pH_apo_-based increase of leaf ABA concentration (Fig. 3) may be the reason for the reduced leaf transpiration rate; that is, the reduction in stomatal conductance (Fig. 4). Our first novel finding is that the pH of the apoplast increases the transcription of *Vp14*, a gene that is key for ABA synthesis in maize. The second novelty is that the functionality of the pH_apo_ transient with respect to reducing the rate of transpiration does not depend on interactions with compounds that are induced by chloride stress. These findings are relevant, since the transient alkalinization of the leaf apoplast is a widespread phenomenon that occurs not only under chloride salinity, but also in response to drought (Bacon *et al.*, 1998) or leaf infections with *Blumeria graminis* (Felle *et al.*, 2008) or *Piriformospora indica* (Felle *et al.*, 2009). Future studies should compare plant species that show the apoplastic alkalization with plant species that do not, in order to understand the underlying mechanism(s) of the transient shift in the apoplastic pH.

## Supplementary data

The following supplementary data are available at *JXB* online.

Table S1. Effect of infiltration on photosynthetic rate.

Table S2. Specificity of the *ZmVp14* primer pair was demonstrated by sequencing the real-time quantitative RT–PCR product.

Table S3. pH_apo_ transient reduces transpiration rate, stomatal conductance, and photosynthetic rate

Fig. S1. Specificity of the *Vp14* primer pair.

eraa589_suppl_Supplementary_Tables_S1-S3_and_Figure_S1

## Data Availability

All data supporting the findings of this study are available within the paper and within its supplementary data published online.

## Abbreviations

ABA: abscisic acid
E: transpiration rate
*g*_s_: stomatal conductance
NCED: 9-*cis*-epoxycarotenoid dioxygenase
OG: Oregon Green
pH_apo_: apoplastic pH
PM: plasma membrane
qRT–PCR: quantitative reverse transcription–PCR
VPD: atmospheric water vapour pressure deficit
