# Supplementary material for: Leaf apoplastic alkalization promotes transcription of the ABA-synthesizing enzyme Vp14 and stomatal closure in Zea mays
Source: J Exp Bot. 2020 Dec 21;72(7):2686–95. doi: 10.1093/jxb/eraa589 (PMC8006549; doi:10.1093/jxb/eraa589)

pH of leaf apoplast is relevant for transcription of ABA-synthesizing key gene *vp14* and for stomatal aperture in *Zea mays* L.

Christoph-Martin Geilfus[1]*, Xudong Zhang[2]*, Axel Mithöfer[3], Lisa Burgel[2,§], Gyöngyi Bárdos[2], Christian Zörb[2]

Supplementary data

Supplementary Table S1. Long-term effect of one-time infiltration on rate of photosynthesis and transpiration. '*Control*', infiltration of 0.4 ml of an aqueous 25 µM OG solution; '*MES pH$_{apo}$ 4.0 (osmotic control)*', infiltration of 0.4 mL of a mixture of 7.5 mM MES at pH 4.7 and 25 µM OG; '*MES pH$_{apo}$ 6.5*', infiltration of 0.4 mL of a mixture of 5 mM MES at pH 6.5 and 25 µM OG; '*MOPSO pH$_{apo}$ 6.5*', infiltration of 0.4 mL of a mixture of 7.5 mM MOPSO at pH 6.5 and 25 µM OG. Mean ± SD of six independent (n = 6) biological replications. Tukey-HSD test revealed no statistical significance ($P \leq 0.05$) between values from all groups, as indicated by ns.

Treatment *(hours after infiltration)*		Transpiration rate *(mmol H_20 m^{-2} s^{-1})*	Photosynthetic rate *(µmol CO_2 m^{-2} s^{-1})*
Control	0 h	5.3±1.2 ^{ns}	9.82±0.7 ^{ns}
	24h	6.0±1.3 ^{ns}	11.42±0.5 ^{ns}
	48h	6.2±1.3 ^{ns}	12.25±0.6 ^{ns}
	72h	5.5±1.5 ^{ns}	10.36±0.4 ^{ns}
MES pH$_{apo}$ 4.0 (osmotic control)	0 h	5.2±1.4 ^{ns}	10.20±0.3 ^{ns}
	24h	5.9±1.2 ^{ns}	11.14±0.8 ^{ns}
	48h	6.0±1.3 ^{ns}	11.91±0.2 ^{ns}
	72h	6.3±1.4 ^{ns}	12.60±0.4 ^{ns}
MES pH$_{apo}$ 6.5	0 h	5.1±1.6 ^{ns}	9.51±0.9 ^{ns}
	24h	5.9±1.0 ^{ns}	11.37±0.6 ^{ns}
	48h	6.1±1.4 ^{ns}	12.08±0.7 ^{ns}
	72h	6.0±1.5 ^{ns}	11.54±0.6 ^{ns}
MOPSO pH$_{apo}$ 6.5	0 h	5.4±1.2 ^{ns}	10.23±0.6 ^{ns}
	24h	5.9±1.4 ^{ns}	11.65±0.5 ^{ns}
	48h	6.0±1.2 ^{ns}	11.79±0.5 ^{ns}
	72h	6.2±1.2 ^{ns}	12.35±0.6 ^{ns}

Supplementary Table S2. Specificity of the *Zmvp14* primer pair was demonstrated by sequencing the real-time quantitative RT-PCR product. Column 1, primer pair. Column 2, DNA sequencing result. Columns 3-7, sequence was alignment against NCBI's reference mRNA sequences (refseq_rna; blastn), search was limited to *Zea mays* L. (taxid:4577). Identical sequencing results were found in our study were identical primer pair was used previous (identical sequencing result shown in the previous work by (Geilfus *et al.*, 2018).

***Zea mays viviparous 14* (*Zmvp14*) primer pair**	**Sequencing summary of real-time quantitative RT-PCR products**<br>(N, any base; primer sequences highlighted grey)	**Significant alignments of sequences**<br>(NCBI blastn on *Zea mays* L. [taxid:4577]; database: refseq_rna)				
		**Genbank sequence ID**	**Description**	**Max score/ Total score**	**Query coverage (%)/ Ident (%)**	**E value**
f 5′–3′<br>TTCTCGGAGGAGGAACAGAGGA<br>r 5′–3′<br>CCAACTGTAACTCTGGTGTGCG	TTTCTCGGAGGAGGAACAGAGGAGCCAGCCATGGATCAGGGGAGAAGTCACCAGAGGGAGCCCAGATCAGTTCCCCGGGGTCTTCNCTGTCNCCNNCNCACNGCACACNAGAGTTACAGTTGGA	NM_001112432.3<br>(No other Blast hits found)	*Zea mays viviparous14* (*vp14*), mRNA	187/187	99/ 94	4e-47

Supplementary Table S3. pH$_{apo}$ transient reduces transpiration rate, stomatal conductance and photosynthetic rate. '*Control*', infiltration of 0.4 ml of an aqueous 25 µM OG solution; *'MES pH$_{apo}$ 4.0 (osmotic control)'*, infiltration of 0.4 mL of a mixture of 7.5 mM MES at pH 4.7 and 25 µM OG; *'MES pH$_{apo}$ 6.5*', infiltration of 0.4 mL of a mixture of 5 mM MES at pH 6.5 and 25 µM OG; *'MOPSO pH$_{apo}$ 6.5*', infiltration of 0.4 mL of a mixture of 7.5 mM MOPSO at pH 6.5 and 25 µM OG. Mean ± SD of six independent (n = 6) biological replications. Statistical significance between values from all groups ($P \leq 0.05$) as calculated by the Tukey-HSD test is indicated by small letters for transpiration rate and capital letters for stomatal aperture.

**Treatment**				**Transpiration rate**	**Stomatal conductance**	**Photosynthetic rate**
Name	*Specification*	*'Minute' on x-axis in Fig. 1*	*Description of time point*	*(mmol H_20 m^{-2} s^{-1})*	*(mmol H_20 m^{-2} s^{-1})*	*(µmol CO_2 m^{-2} s^{-1})*
Control	Start, before infiltration	10	No alkalisation, no ABA accumulation	5.3±1.4^{a}	252±42$^{\alpha}$	10.3±0.8$^{€}$
	60 min after infiltration	100	No alkalisation, no ABA accumulation	5.5±1.4^{a}	269±45$^{\alpha}$	10.5±0.5$^{€}$
	After re-acidification	200	No alkalisation, no ABA accumulation	5.4±1.4^{a}	262±40$^{\alpha}$	10.5±0.4$^{€}$
	2 h after re-acidification	320	No alkalisation, no ABA accumulation	5.8±1.1^{a}	283±45$^{\alpha}$	11.2±0.4$^{€}$
	4 h after re-acidification	440	No alkalisation, no ABA accumulation	6.1±0.9^{a}	294±48$^{\alpha}$	12.1±0.2$^{₱}$
MES pH$_{apo}$ 4.0 (osmotic control)	Start, before infiltration	10	No alkalisation, no ABA accumulation	5.0±1.3^{a}	244±41$^{\alpha}$	10.1±0. 5$^{€}$
	60 min after infiltration	100	No alkalisation, no ABA accumulation	5.4±1.5^{a}	259±39$^{\alpha}$	10.4±0.6$^{€}$
	After re-acidification	200	No alkalisation, a few ABA accumulated	5.4±1.2^{a}	264±43$^{\alpha}$	10.2±0.7$^{€}$
	2 h after re-acidification	320	No alkalisation, no ABA accumulation	5.4±1.3^{a}	267±46$^{\alpha}$	10.5±0.3$^{€}$
	4 h after re-acidification	440	No alkalisation, no ABA accumulation	4.7±1.1^{a}	230±38$^{\alpha}$	8.3±0.6$^{฿}$
MES pH$_{apo}$ 6.5	Start, before infiltration	10	Before alkalisation, no ABA accumulation	5.0±1.3^{a}	242±41$^{\alpha}$	9.9±0.7$^{€}$
	60 min after infiltration	100	Apoplast alkalinised, no ABA accumulation	5.4±1.3^{a}	256±44$^{\alpha}$	10.4±0.3$^{€}$
	After re-acidification	200	Apoplast re-acidified, ABA accumulated	4.7±1.6^{a}	227±39$^{\alpha}$	8.5±0.3$^{฿}$
	2 h after re-acidification	320	Apoplast re-acidified, ABA accumulated	2.3±0.4^{b}	128±25$^{\beta}$	5.2±0.3$^{¥}$
	4 h after re-acidification	440	Apoplast re-acidified, ABA was not measured	1.8±0.3^{bc}	97±26 $^{\beta\gamma}$	3.1±0.2$^{£}$
MOPSO pH$_{apo}$ 6.5	Start, before infiltration	10	Before alkalisation, no ABA accumulation	5.5±1.5^{a}	267±43$^{\alpha}$	10.6±0.4$^{€}$
	60 min after infiltration	100	Apoplast alkalinised, no ABA accumulation	5.4±1.2^{a}	258±37$^{\alpha}$	10.5±0.4$^{€}$
	After re-acidification	200	Apoplast re-acidified, ABA accumulated	4.8±1.5^{a}	235±40$^{\alpha}$	8.4±0.3$^{฿}$
	2 h after re-acidification	320	Apoplast re-acidified, ABA accumulated	1.7±0.1^{c}	85±14$^{\gamma}$	2.9±0.1$^{£}$
	4 h after re-acidification	440	Apoplast re-acidified, ABA was not measured	1.9±0.3^{bc}	102±26 $^{\beta\gamma}$	3.1±0.1$^{£}$

Supplementary Fig. S1. Specificity of the *vp14* primer pair. Real-time qRT-PCR products were separated on agarose gels. Only one single DNA band appeared. *In-silico* analysis using the Primer-BLAST software predicted a band size of ~ 117 bp. Gels contain 2% agarose, Tris-borate-EDTA, 5 µl SYBR Safe pro 100 ml Gel (SYBR® Safe DNA Gel Stain, Invitrogen).

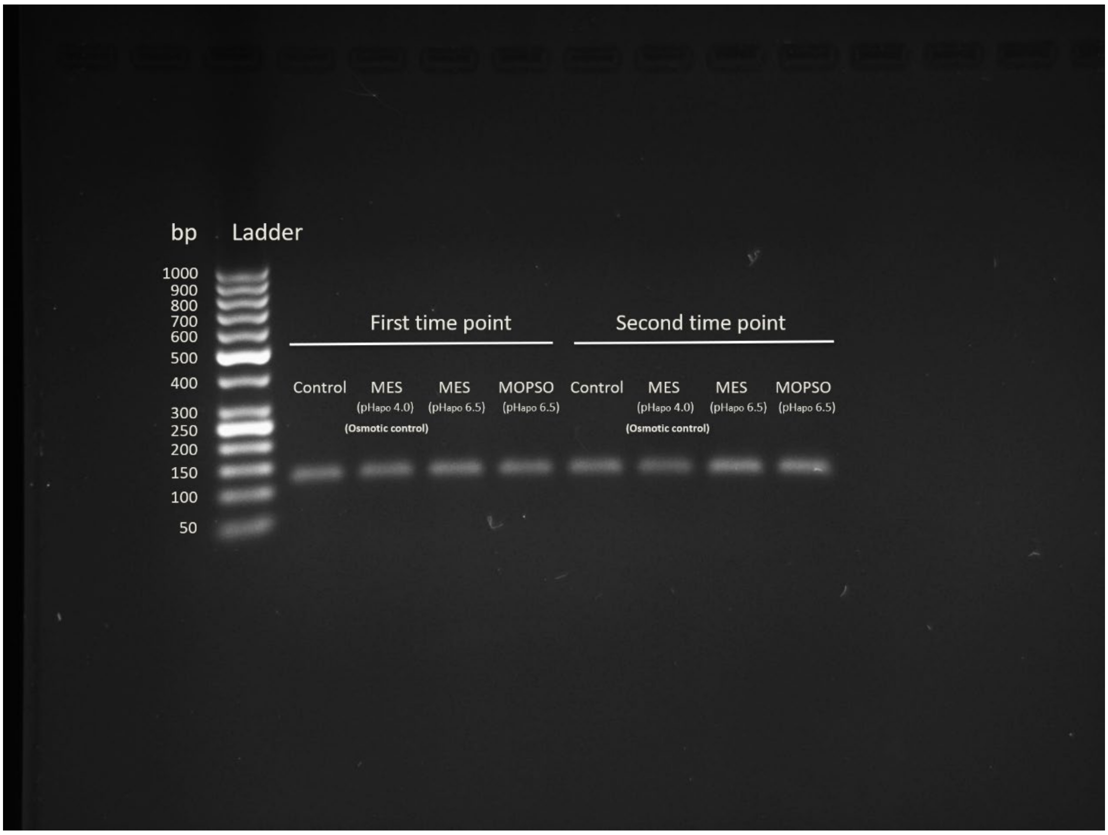

Supplement: eraa589_suppl_Supplementary_Tables_S1-S3_and_Figure_S1 [file eraa589_suppl_supplementary_tables_s1-s3_and_figure_s1.pdf]
